# Recognising serous atrophy of bone marrow: a review of imaging findings

**DOI:** 10.1007/s00256-025-04963-w

**Published:** 2025-06-20

**Authors:** Tania Toma, Stuart Viegas, Dimitri Amiras, Christopher Lord

**Affiliations:** 1 Department of Radiology, Imperial College NHS Healthcare Trust, Praed Street, London, W2 1NY UK; 2 Department of Neurology, Imperial College NHS Healthcare Trust, Praed Street, London, W2 1NY UK; 3 Department of Surgery & Cancer - Faculty of Medicine, Imperial College, London, UK

**Keywords:** Serous atrophy, Bone marrow, MRI, Differential diagnosis

## Abstract

Serous atrophy of bone marrow (SABM) is a rare bone marrow disorder associated with severe illness and poor nutritional state. This results in characteristic MRI signal alterations of the bone marrow and surrounding soft tissues. The unusual imaging features are often misinterpreted as technical error or erroneously attributed to underlying malignancy, resulting in unnecessary additional imaging. Therefore, it is important to differentiate SABM from technical failures and other infiltrating marrow processes. This review provides an overview of the imaging characteristics of SABM and associated imaging pitfalls, based on examples from our own practice.

## Introduction

Serous atrophy of bone marrow (SABM), also known as gelatinous transformation of bone marrow, is a disorder characterised by diffusely abnormal bone marrow signal and subcutaneous adipose tissue changes. In histological samples of bone marrow, hallmark features include hypoplasia of adipocytes, a reduction of hematopoietic elements, and deposition of extracellular gelatinous substances. SABM is a non-specific indicator of severe illness but is generally associated with prolonged malnutrition states (such as anorexia or cachexia). Although considered a rare condition, the reported incidence of SABM has gradually increased, ranging from 0.2 to 4.8% in more recent studies [1, 2]. SABM results in characteristic imaging findings on magnetic resonance imaging (MRI) including diffuse hypointensity of bone marrow on T1-weighted images and hyperintensity on T2-weighted and fluid-sensitive sequences. Signal abnormalities are also demonstrated in the adjacent soft tissues with characteristic hypointense signal on T1-weighted images and hyperintense signal on fluid-sensitive sequences. These unusual findings are often attributed to technical factors, such as incomplete fat suppression, Dixon fat/water swaps, or misinterpreted for neoplastic conditions. As a result, patients are often subjected to unnecessary additional imaging and investigations before a diagnosis of SABM is made. This review presents an update on the clinical and imaging features of SABM based on a review of the literature and examples from our own practice.

## Pathophysiology

The pathogenesis of SABM is not fully understood; however, it is thought to represent a state of severe catabolism, causing a depletion of adipocytes in subcutaneous and visceral fat compartments. Initially, there is a paradoxical increase in marrow fat, thought to be due to the preferential differentiation of mesenchymal stem cells into adipocytes rather than osteoblasts. Eventually, these bone marrow fat stores are replaced by gelatinous substances in the bone marrow stroma, including acid mucopolysaccharides and hyaluronic acid, which both inhibit the growth of erythropoietic tissue [2–4]. It is hypothesised that the excess deposition of these gelatinous substances creates a microenvironment hostile for haematopoietic proliferation, with a resultant reduction in bone marrow haematopoietic cells [3, 5]. In addition to the presence of extracellular gelatinous substances, histological appearances of bone marrow include hypocellularity of the hematopoietic cells and scant adipocytes. Due to the hypocellular marrow appearances in SABM, the microscopic findings can have a pathological differential diagnosis that includes bone marrow edema, necrosis, amyloid, or aplastic anemia. To aid differentiation between these entities, Alcian blue staining can be utilised which stains blue in SABM due to the presence of the extracellular mucopolysaccharide-rich gelatinous substances deposited in bone marrow. Other hypocellular marrow conditions lack the presence of these gelatinous materials and therefore Alcian blue staining remains negative in these processes [2, 6].

## Clinical presentation

SABM is a non-specific indicator of severe illness and generally a marker of poor nutritional status but can be associated with a spectrum of underlying diseases. The most common associations include anorexia nervosa, malnutrition, or malabsorption with weight loss. Other causes include alcohol excess, congestive cardiac failure (CCF), chronic infection such as TB, chronic inflammatory disease such as SLE, and metabolic disorders including adrenal insufficiency [2, 4]. Historically, there have been published reports of SABM in patients with late-stage HIV infection [7, 8], however with the advent of anti-retroviral therapies and reduced prevalence of advanced disease, this is likely no longer a common entity. A study of 155 patients by Bohm et al. suggests a bimodal distribution of SABM with the aetiologies varying according to the age of the patient. Anorexia nervosa and severe chronic infection were most prevalent in younger patients with SABM, whereas CCF and lymphoma were the main causes of SABM in patients above forty years old [4].

The clinical findings of SABM are non-specific and include weight loss, anaemia (seen in 78% and 81% of patients with SA respectively) and cachexia. Fevers are also a common finding. While a chronic febrile state may not directly contribute to SABM, it is frequently present in the context of catabolic states, chronic inflammation and infection, which are associated with SABM [4]. Muscle symptoms in SA, including pain and weakness, have been described in case reports [9]. Another important complication in patients with SABM is the development of fractures due to a reduction in underlying bone quality [10]. Despite a relatively clear clinical phenotype of weight loss and cachexia, SABM is usually an incidental finding detected on imaging. In fact, in most patients, SABM is not suspected prior to imaging and the index MRI is requested for other reasons, most commonly pain associated with fractures, followed by evaluation of soft tissue ulceration [11]. Therefore, communication with the clinical team is beneficial to help identify patients at risk of SABM and optimise the interpretation of their imaging.

## Imaging features

### MRI

**Fig. 1:**
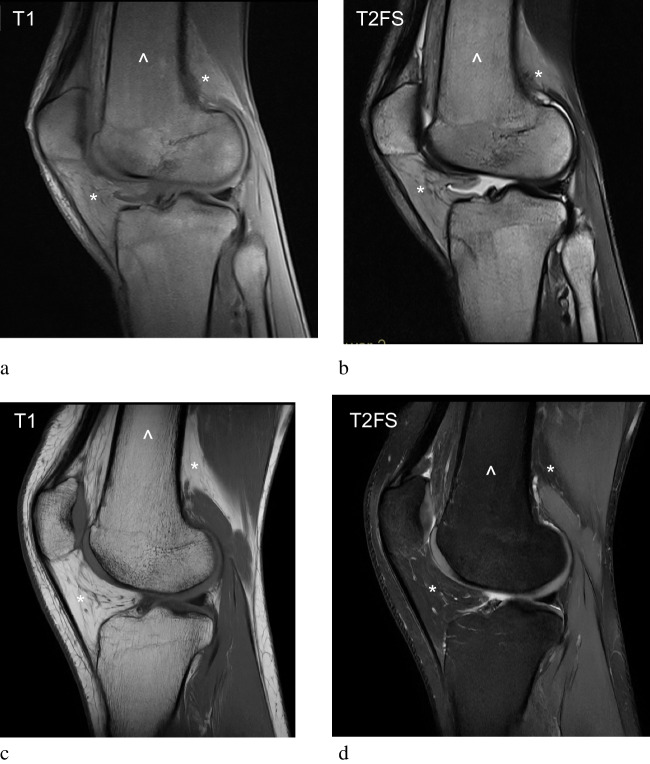
SABM in a 53-year-old female with a body mass index (BMI) of 16 kg/m^2^ due to excessive exercise. Sagittal T1 weighted (**a**) and T2 fat-saturated (T2 FS) (**b**) sequences of the knee demonstrate diffuse hypointense marrow signal on T1 and mild hyperintensity on T2 FS (arrowheads). Characteristic signal abnormalities are also demonstrated in the adjacent subcutaneous tissues (asterisks), with hypointense signal on T1-weighted images and hyperintense signal on fluid-sensitive sequences, giving rise to the inverted signal characteristics of SABM. Sagittal T1 weighted (**c**) and T2 FS (**d**) sequences of a normal knee for comparison, demonstrating normal marrow signal (arrowheads) and subcutaneous fat (asterisks)

**Fig. 2:**
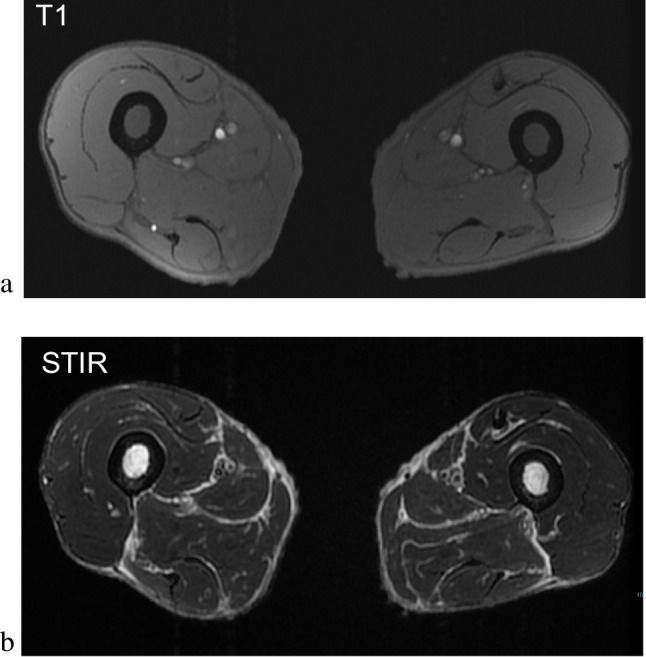
SABM in a 45-year-old male with anorexia nervosa and rapid weight loss. T1-weighted sequences (**a**) and STIR sequences (**b**) demonstrate diffuse loss of the subcutaneous and intermuscular fat in SABM. Residual visible fat shows abnormal intermediate to hypointense T1 signal and hyperintense fluid sensitive signal. Hypointense T1-weighted marrow signal in (**a**) and STIR hyperintense marrow signal in (**b**) is also consistent with SABM

SABM produces characteristic MRI findings, typically presenting as diffuse mild hypointensity of the bone marrow on T1-weighted images and hyperintensity on T2-weighted or fluid sensitive sequences, relative to muscle [11]. Patients with SABM also exhibit widespread fat depletion due to underlying malnutrition or severe systemic disease. This affects the subcutaneous tissues, which are not only reduced in volume, but demonstrate abnormal hypointensity on T1-weighted sequences and high signal on fluid-sensitive sequences. It is this combination of signal abnormalities in both the bone marrow and subcutaneous tissues which are colloquially referred to as the ‘flip-flop’ effect within the literature (Fig. 1). The absence of fat stores in SABM and resultant increase in marrow water content is thought to explain the non-visualisation of fatty bone marrow on T1-weighted images and apparent failure of fat saturation in subcutaneous tissues on fluid-sensitive images. Explanations for the signal changes in subcutaneous tissues include increased loose vascular tissue relative to adipose tissue, increased vascular permeability, and hypoalbuminaemia [12]. These signal abnormalities are not confined to subcutaneous tissues but are also demonstrated in both intermuscular and visceral fat compartments, where there is often a lack of fat and abnormal hyperintense signal on fluid sensitive sequences [13, 14] (Figs. 2, 6c and d).

**Fig. 3:**
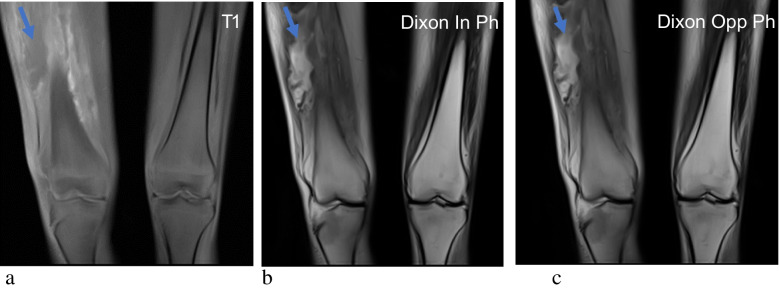
SABM in a 35-year-old female with a background of diabetes mellitus and myonecrosis. T1-weighted image (**a**) demonstrates the characteristic intermediate to mildly hypointense marrow signal in SABM. No visible difference in bone marrow signal between the coronal T2 Dixon in-phase (**b**) and opposed-phase images (**c**), resulting in nearly identical images and minimal chemical shift. An intramuscular haematoma was also demonstrated in the vastus lateralis (blue arrows)

Dixon sequences can be utilised to demonstrate the lack of marrow adiposity in patients with SABM, by quantifying the fat depletion and water predominance seen. Due to the paucity of fat, there is no visible difference in bone marrow signal between the opposed-phase and in-phase images, resulting in nearly identical images and minimal chemical shift artefact [15, 16] (Fig. 3). There also appears to be a positive correlation between T1 and T2 relaxation times and disease severity (quantified by body mass index, neutrophil level, haemoglobin level, leukocyte level and red cell levels). For example, Vande Berg et al. demonstrated greater T1 and T2 prolongation in bone marrow (and therefore increased T1 hypointensity and T2 hyperintensity) in patients with a lower body mass index [17].

**Fig. 4:**
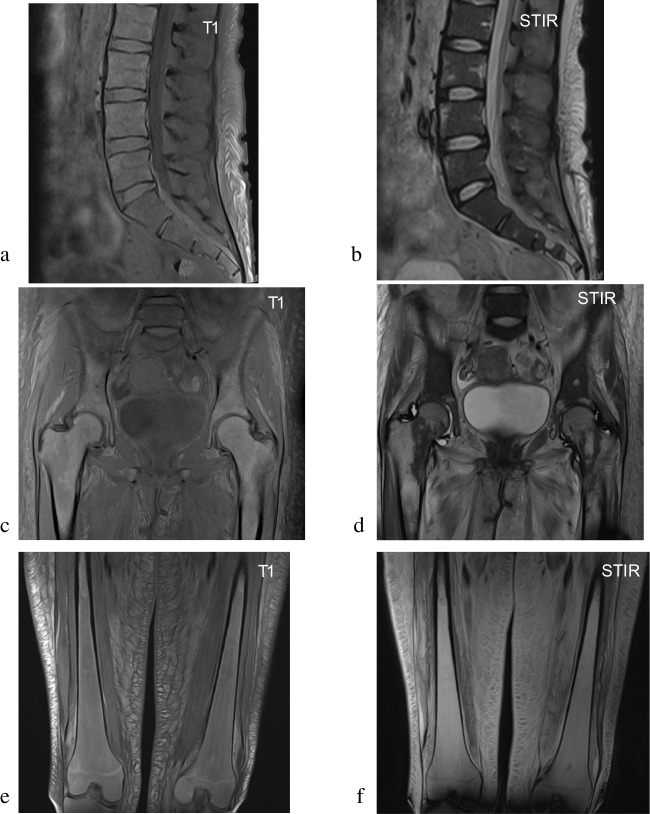
SABM in a 32-year-old female with Type 1 diabetes mellitus as well as weight loss and malnutrition secondary to autonomic diarrhoea. The patient underwent MRI due to a persistent staphylococcus aureus bacteraemia and lower back pain. This figure illustrates the pattern of involvement of SABM with central sparing. T1-weighted images of the lumbar spine and proximal pelvis (**a**, **c**) show diffuse changes in the axial skeleton with relative preservation of normal marrow signal on fluid sensitive sequences (**b**, **d**). There is a transition in appearances on the fat-saturated sequences at the proximal femoral level. Coronal images of the distal femur in the same patient now demonstrate diffuse marrow changes on both T1-weighted (**e**) and fluid-sensitive sequences (**f**)

Another useful imaging feature in patients with SABM is the characteristic distribution of bone marrow signal changes. SABM preferentially involves the appendicular skeleton rather than flat bones, typically beginning in the distal extremities and progressing centrally. This is thought to replicate the pattern of haematopoietic to fatty marrow conversion in the early years of life. As a result, there is often relative preservation of marrow signal in the spine or pelvis, with diffuse changes in the appendicular skeleton (Fig. 4). This distribution is converse to many bone marrow disorders which usually commence in the axial skeleton and migrate distally [18]. If signal abnormalities in patients with SABM involve the axial skeleton or pelvis, this generally indicates more extensive and severe disease [19]. In addition, the distribution of signal abnormality is usually more diffuse rather than focal [17, 20], although there is a case study that suggests early SABM can present as a smaller focus of signal change before becoming more widespread [13].

One difficulty in patients presenting with SABM is the identification of fractures. Patients with SABM are prone to fractures, partly due to their underlying physiological condition as well as their altered bone marrow composition, resulting in reduced biomechanical bone strength. Boutin et al. found 47% of patients with SABM sustained concurrent stress fractures [11]. However, multiple studies have highlighted the difficulty in detecting fractures on MR in this cohort, which are often occult due to the diffusely abnormal background bone marrow changes. The lack of fat saturation in the marrow on fluid-sequences and decreased signal on T1 images can often mask bone marrow edema as well as subtle trabecular fractures [21, 22]. Therefore, an increased index of suspicion is required to identify subtle fractures in this vulnerable population and targeted plain radiographs or CT can be used if there is uncertainty.

### Other imaging modalities

**Fig. 5:**
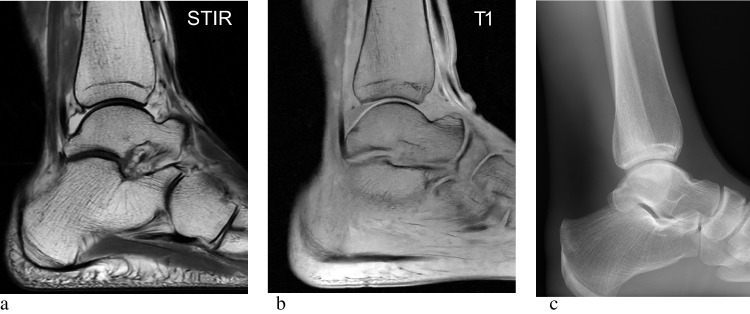
SABM in a 32-year-old female with a low BMI secondary to anorexia nervosa. T1-weighted images show mildly hypointense signal of the subcutaneous tissues (**a**). Short inversion time inversion recovery (STIR) sequence of the ankle (**b**) demonstrates reduced thickness and abnormal hyperintensity of the subcutaneous adipose tissues. The lack of adipose tissue and loss of fat planes is also demonstrated on the corresponding plain radiograph (**c**)

**Fig. 6:**
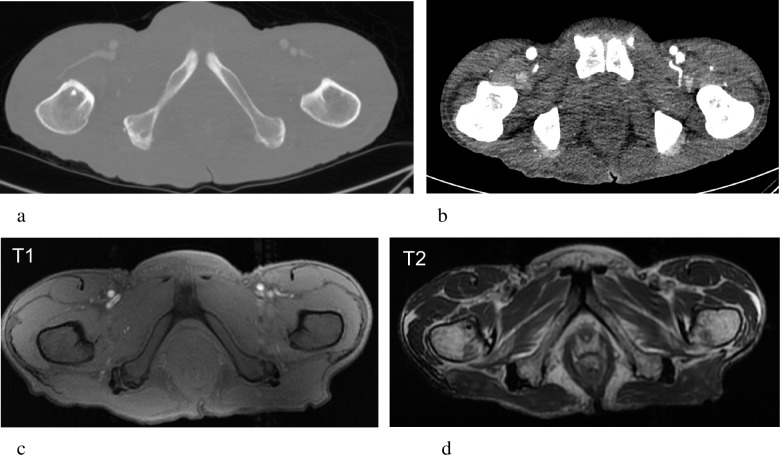
SABM in a 45-year-old male presenting with rapid intentional weight loss, cachexia and deranged liver function on admission. Axial CT image with bone windowing shows normal bone texture, cortical thickness and density (**a**). Portal-venous phase axial CT image demonstrates a paucity of pelvic fat on the soft tissue windows (**b**). Diffuse hypointensity of bone marrow on the T1-weighted image (**c**) and hyperintensity on T2 FS (**d**) is typical of SABM. Residual visible fat shows abnormal intermediate to hypointense T1 signal and hyperintense fluid sensitive signal. A marked paucity of pelvic fat is also noted

There is limited evidence to support the diagnosis of SABM based solely on other imaging modalities other than MRI. Plain radiography, computed tomography (CT) and positron emission tomography-computed tomography (PET-CT) typically demonstrate normal osseous appearances or osteopenia. However, ancillary features, particularly within the intermuscular and subcutaneous tissues, may provide indirect clues to the presence of SABM. For example, both plain radiographs and CT images may demonstrate a generalised reduction or absence of adipose tissue (Figs. 5, 6b) [13]. Due to the marrow signal abnormalities in SABM, many of these patients undergo PET-CT to identify a hidden underlying malignancy; however, PET-CT usually fails to identify a hypermetabolic focus within the bone marrow in patients with SABM [13, 23]. There is only a single case in the literature reporting a focal area of marrow FDG-PET uptake that revealed SABM after histochemical analysis from bone biopsy [24].

Single-voxel MRI spectroscopy has been proposed as a problem-solving technique with lipids representing the smallest peak and water representing the largest peak in SABM patients. In patients without SABM, there is normally a small water peak and a profound lipid peak [15]. For example, Bredella et al. demonstrated that patients with anorexia nervosa had higher levels of marrow fat quantified by 1H-magnetic resonance spectroscopy in the lumbar vertebrae and femur compared to controls. Conversely, these patients had lower bone mineral density values measured by dual-energy x-ray absorptiometry and lower total abdominal adipose tissue [25].

## Differential diagnoses and imaging pitfalls

The unusual imaging features of SABM are often misinterpreted as technical error or attributed to other causes of diffuse bone marrow signal abnormality, including bone marrow reconversion, bone marrow infiltration, infection, and radiation induced changes [12].

### Marrow reconversion

**Fig. 7:**
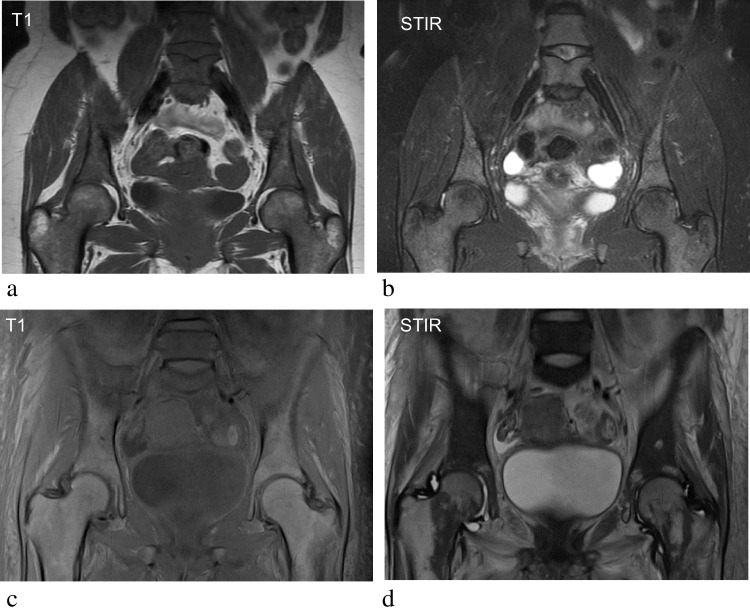
Bone marrow reconversion in a 56-year-old female with sickle cell disease. Sagittal T1-weighted image (**a**) demonstrates diffuse hypointense marrow signal in the pelvis and proximal femora with corresponding mild hyperintensity on T2 fat-saturated sequences (**b**). Typical H-shaped vertebrae secondary to sickle cell disease are also demonstrated. In contrast to SABM, there is normal T1-weighted signal of the subcutaneous tissues (**a**) and expected suppression of fat on the STIR sequences (**b**). Therefore, bone marrow reconversion is an important differential diagnosis of diffuse bone marrow signal changes, but without the characteristic soft tissue signal abnormalities seen in SABM. Sagittal T1-weighted (**c**) and T2-weighted fat-saturated sequences (**d**) in SABM from Fig. 4 for comparison, demonstrating characteristic inverted signal changes of the bone marrow with diffuse signal changes in the subcutaneous tissues

The typical distribution of SABM also differs from bone marrow reconversion, which is another condition resulting in diffuse low T1-weighted and hyperintense fluid-sensitive bone marrow signal. This phenomenon is often observed in athletic patients with significant oxygen demand but can also be seen in obese patients, diabetics, smokers, and those with conditions related to anemia. Marrow reconversion begins in the axial skeleton where there is a higher concentration of red marrow and extends peripherally [26]. However, as previously discussed, SABM usually originates in the distal skeleton where fatty marrow predominates. Furthermore, unlike SABM, marrow reconversion demonstrates normal T1-weighted signal of the subcutaneous tissues with expected suppression of subcutaneous fat on fluid-sensitive sequences (Fig. 7).

### Malignancy

**Fig. 8:**
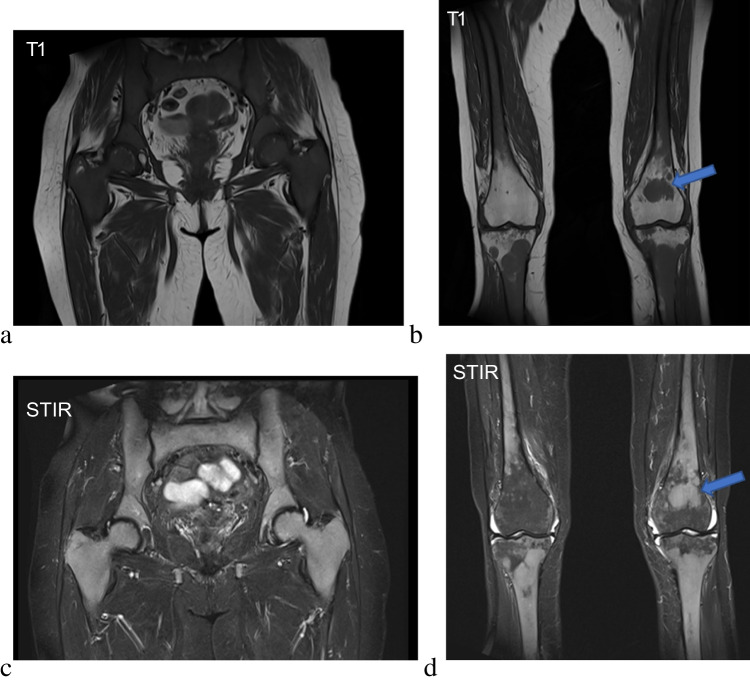
Seventy-three-year-old female with myelodysplastic syndrome. T1-weighted images of the pelvis and femora (**a**, **b**) demonstrate diffuse infiltrative appearances of the marrow with corresponding hyperintensity of the marrow on STIR sequences (**c**, **d**). Unlike SABM, the adjacent subcutaneous tissues are not involved in malignancy and are appropriately fat suppressed on fluid-sensitive sequences. Therefore, malignancy represents an important differential diagnosis of diffuse bone marrow signal change, but without the characteristic soft tissue signal abnormalities seen in SABM. The axial distribution is also suggestive of malignancy rather than SABM. The focal lesion in the left distal femoral medullary cavity (arrowed) is focal in nature, which is more in keeping with malignant infiltration, compared to SABM where changes are more diffuse

This distribution of SABM is also a differentiating factor between SA and malignancy, with malignant marrow infiltration primarily affecting the proximal and axial skeleton where there is pre-existing red marrow. Malignant lesions are also often focal, rounded in morphology, and may be multifocal, in contrast to the diffuse appearance seen in SABM [11] (Fig. 8). The administration of intravenous contrast in MR can aid differentiation between SABM and neoplastic or infective aetiologies, since SABM is reported to show a lack of enhancement in both bone marrow and subcutaneous tissues [11, 13]. The absence of signal abnormalities in the adjacent subcutaneous fat is another key differentiating factor between malignancy and SABM.

### Incomplete fat suppression

**Fig. 9:**
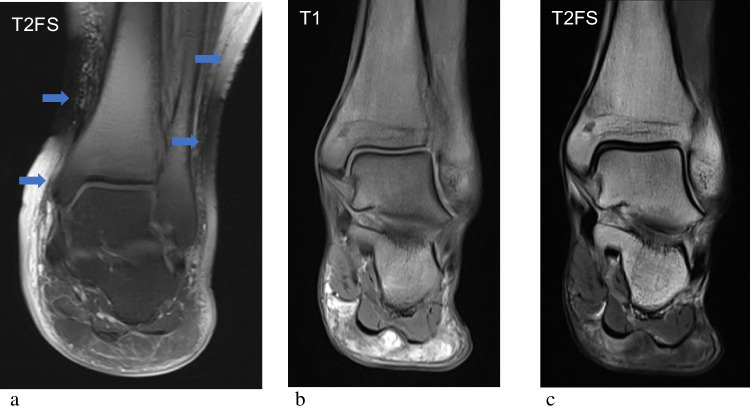
Incomplete fat suppression in a 62-year-old male in (**a**). Coronal fat-saturated T2 weighted image of the ankle demonstrates diffuse bone marrow hyperintensity as well as hyperintensity of the subcutaneous tissues, mimicking the appearances of SABM. Alternating bands of high and low signal in a non-anatomical pattern (blue arrows) is typical of failed fat suppression. Coronal T1 weighted (**b**) and T2 weighted fat-saturated sequences (**c**) in SA for comparison, demonstrate homogenous signal changes in the bone marrow (diffuse T1 hypointensity and T2 hyperintensity) and soft tissues, but without the band-artefact seen in incomplete fat suppression

**Fig. 10:**
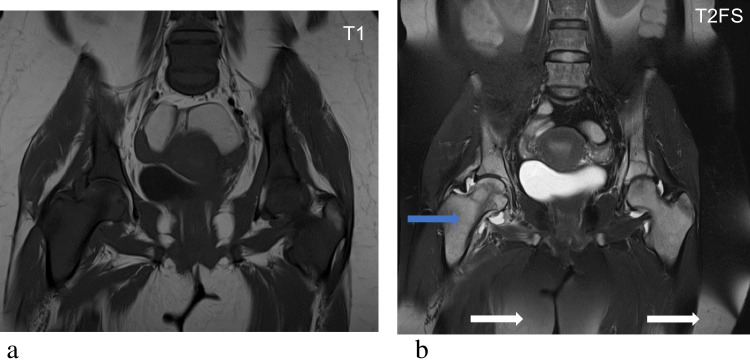
Acute leukaemia in a 32-year-old female with incomplete fat suppression on fluid sensitive sequences. Coronal T1-weighted image (**a**) of the pelvis, lumbar spine and proximal femora demonstrate diffuse hypointensity of bone marrow in keeping with marrow infiltration. Corresponding T2-fat saturated sequences (T2 FS) (**b**) demonstrate diffuse hyperintensity of bone marrow with serpiginous areas of signal abnormality in the femoral heads and adjacent acetabula, suspicious for osteonecrosis. There are also peripheral band-like signal changes that do not persist on the T1-weighted images, in keeping with incomplete fat saturation. Therefore, the T2 FS image mimics the inverted signal changes of SABM with hyperintense signal changes in the marrow (blue arrow) and subcutaneous tissues (white arrows)

Incomplete fat suppression describes the presence of unwanted high signal on fluid sensitive sequences in areas where signal from fat should be suppressed. This can manifest as patchy areas of bright signal or the appearance of alternating bright and dark bands that do not follow an anatomical pattern. These artefactual signal patterns can mimic true edema or pathology. Incomplete fat suppression occurs due to a variety of factors including magnetic field inhomogeneities, chemical shift artefacts and the presence of metallic implants [27]. The hyperintense signal of bone marrow, subcutaneous and visceral fat in studies with incomplete fat suppression produces similar imaging appearances to SABM, resulting in confusion between the two entities (Figs. 9, 10). Boutin et al. reported that 23% of patients required unnecessary repeat imaging due to misinterpretation of marrow signal in SABM as incomplete fat suppression. To avoid this pitfall, both fat-suppressed T2-weighted and STIR sequences should be acquired, as STIR is less sensitive to magnetic field variation. Furthermore, SABM produces a more homogenous pattern of signal abnormality, whilst incomplete fat suppression often results in patchy or banded signal inconsistent with underlying anatomy [11]. Given the overlap in features, increasing awareness of SABM amongst MRI technologists may help prompt radiologist input before unnecessary repeated imaging is performed.

## Treatment/outcomes

The long-term outcomes and prognosis of SABM are poorly documented in the literature. The presence of SABM is not believed to carry any independent prognostic significance, and outcomes are more dependent on the nature and severity of the underlying disease [2, 4]

In some cases, SABM can be reversed if the underlying condition can be treated successfully, for example in anorexia or malnutrition, with several studies reporting the reversal of SABM following normalisation of nutritional status [28–30]. However, when SABM presents in the setting of advanced cachexia, such as in malignancy or chronic infection, treatment of the underlying cause is more challenging, and is associated with poorer outcomes. For example, Boutin et al. demonstrated that patients with SABM and cancer had a particularly poor prognosis with survival rates of less than two months following the diagnosis of SABM [11]. The use of granulocyte colony stimulating factor (G-CSF) to stimulate proliferation of granulocytic precursors has been used to reverse SABM, but has only been successfully demonstrated in a handful of cases [29, 31].

## Conclusion

SA is an under-recognised condition resulting in diffuse bone marrow signal abnormality. We present the pertinent imaging findings of SA, illustrated by examples from our experience. Serous atrophy is an indicator of severe underlying illness and is often an incidental finding on MRI. SA should be considered in the presence of diffusely hypointense bone marrow on T1-weighted sequences and hyperintense marrow on fluid sensitive sequences, especially in the context of severe malnutrition or chronic disease. These appearances can be misinterpreted as technical error or myeloproliferative disorders; therefore, early recognition of SA is the key to prevent unnecessary additional imaging and investigations.

## Data Availability

Image data was extracted from our clinical PACS and stored in DICOM standard format. Raw image data is not publicly available to preserve individuals’ privacy under the UK General Data Protection Regulation (UK GDPR) and the Data Protection Act 2018.
